# Impact of Sarcopenia on Prognosis in Primary Hepatocellular Carcinoma Patients Treated with Transcatheter Arterial Chemoembolization: A Single Center Retrospective Study

**DOI:** 10.7150/jca.92976

**Published:** 2024-02-04

**Authors:** Yaowei Bai, Jiacheng Liu, Ying Wang, Binqian Zhou, Xiaoming Liu, Xiangjun Dong, Chuansheng Zheng

**Affiliations:** 1 Department of Radiology, Union Hospital, Tongji Medical College, Huazhong University of Science and Technology, Wuhan 430022, China.; 2 Hubei Province Key Laboratory of Molecular Imaging, Wuhan 430022, China.; 3 Department of Radiology, Tongren Hospital of Wuhan University (Wuhan Third Hospital), Wuhan University, Wuhan 430060, China.; 4 Department of Ultrasound, The Central Hospital of Wuhan, Tong ji Medical College, Huazhong University of Science and Technology, Wuhan 430014, China.; Yaowei Bai, Jiacheng Liu and Ying Wang contributed equally.

**Keywords:** sarcopenia, transcatheter arterial chemoembolization, hepatocellular carcinoma, prognosis

## Abstract

**Objective:** This study aimed to investigate the prognostic effect of sarcopenia on primary hepatocellular carcinoma (HCC) patients after transcatheter arterial chemoembolization (TACE).

**Methods:** This retrospective study enrolled 265 patients diagnosed with HCC who underwent TACE between April 2014 and February 2021. The patients were divided into two groups: the sarcopenia group (n=133) and the non-sarcopenia group (n=132). The study analyzed the differences in overall survival (OS) and progression-free survival (PFS) using Kaplan-Meier curves. The independent risk factors for OS and PFS were determined using univariate and multivariate Cox regression analysis. Based on these factors, the study constructed a prognostic risk grading system.

**Results:** At 3 and 6 months post-TACE, the prognoses of the sarcopenia group were worse than that of the non-sarcopenia group according to the mRECIST criteria. Kaplan-Meier curves showed that the cumulative OS and PFS rate in the non-sarcopenia group were significantly higher compared to the sarcopenia group (HR=3.319, 95%CI: 2.283-4.824, Log-rank *P* < 0.001; HR=0.631, 95%CI: 0.486-0.820, Log-rank *P* < 0.001). Sarcopenia, maximal tumor diameter, and AFP ≥ 200 ng/mL were independent risk factors for OS and PFS. The prognostic risk grading system based on sarcopenia, AFP ≥ 200 ng/mL, and maximal tumor diameter≥8.9 cm showed significant differences in prognosis between risk groups.

**Conclusion:** Sarcopenia had excellent predictive value for OS and PFS in patients after TACE, and AFP ≥ 200 ng/mL and maximal tumor diameter were also independent risk factors for a poor prognosis. The prognostic risk grading system based on sarcopenia, AFP, and maximal tumor diameter had good guiding value for the prognosis of patients.

## Introduction

Sarcopenia is characterized by a decline in skeletal muscle volume and function, which has been shown to occur in various malignant tumors including hepatocellular carcinoma (HCC), lung cancer, and gastric cancer^1^-^3^. The prevalence of sarcopenia varied widely across studies^4^, ^5^. A recent meta-analysis showed that the pooled prevalence of sarcopenia was 39% (95% CI: 33-45%) (n = 8203)^6^. Sarcopenia is a significant factor in the prognosis of liver cancer patients^4^, and studies have demonstrated its association with poor clinical outcomes in patients treated with sorafenib or lenvatinib^7^-^9^. However, existing clinical staging and prognostic scoring systems, such as the albumin bilirubin (ALBI), Child-Pugh score, and model for end-stage liver disease (MELD) score, fall short of providing a comprehensive evaluation of patients' nutritional status or the intricate relationships among liver cirrhosis, HCC, and sarcopenia. Although the updated Barcelona clinic liver cancer (BCLC) staging system included performance status (PS) in prognosis evaluation^10^, it still cannot evaluate the nutritional and functional status of patients, and the evaluation of PS has reporting bias and remains controversial^11^. Therefore, establishing a prognostic evaluation system that includes sarcopenia would benefit the treatment and prognosis of patients.

Transcatheter arterial chemoembolization (TACE) is the primary treatment for unresectable HCC, and its basic principle is to maintain high concentrations of chemotherapy drugs in the tumor site to maximize the killing effect of chemotherapy drugs on tumors and reduce systemic adverse reactions^12^, ^13^. Previous studies have confirmed the relationship between post-TACE sarcopenia progression and poor clinical outcomes in patients with HCC^14^, ^15^. However, researches on the impact of pre-TACE sarcopenia on the prognosis of HCC patients after TACE treatment are still insufficient and there is controversy about the prognostic value of pre-TACE sarcopenia^16^, ^17^. Therefore, our study aimed to analyze the impact of pre-TACE sarcopenia on the overall survival (OS) and progression-free survival (PFS) of HCC patients who were treated with TACE. Additionally, we aimed to construct a TACE prognostic risk grading system to guide clinical treatment and improve patient prognosis.

## Materials and methods

This study was approved by the Institutional Review Board (IRB) of Wuhan Union Hospital, Tongji Medical College, Huazhong University of Science and Technology and was conducted according to the tenets of the 1975 Declaration of Helsinki^18^. Written informed consent was revoked for this retrospective study by IRB of Wuhan Union Hospital, Tongji Medical College, Huazhong University of Science and Technology.

### Patients

**Figure 1:**
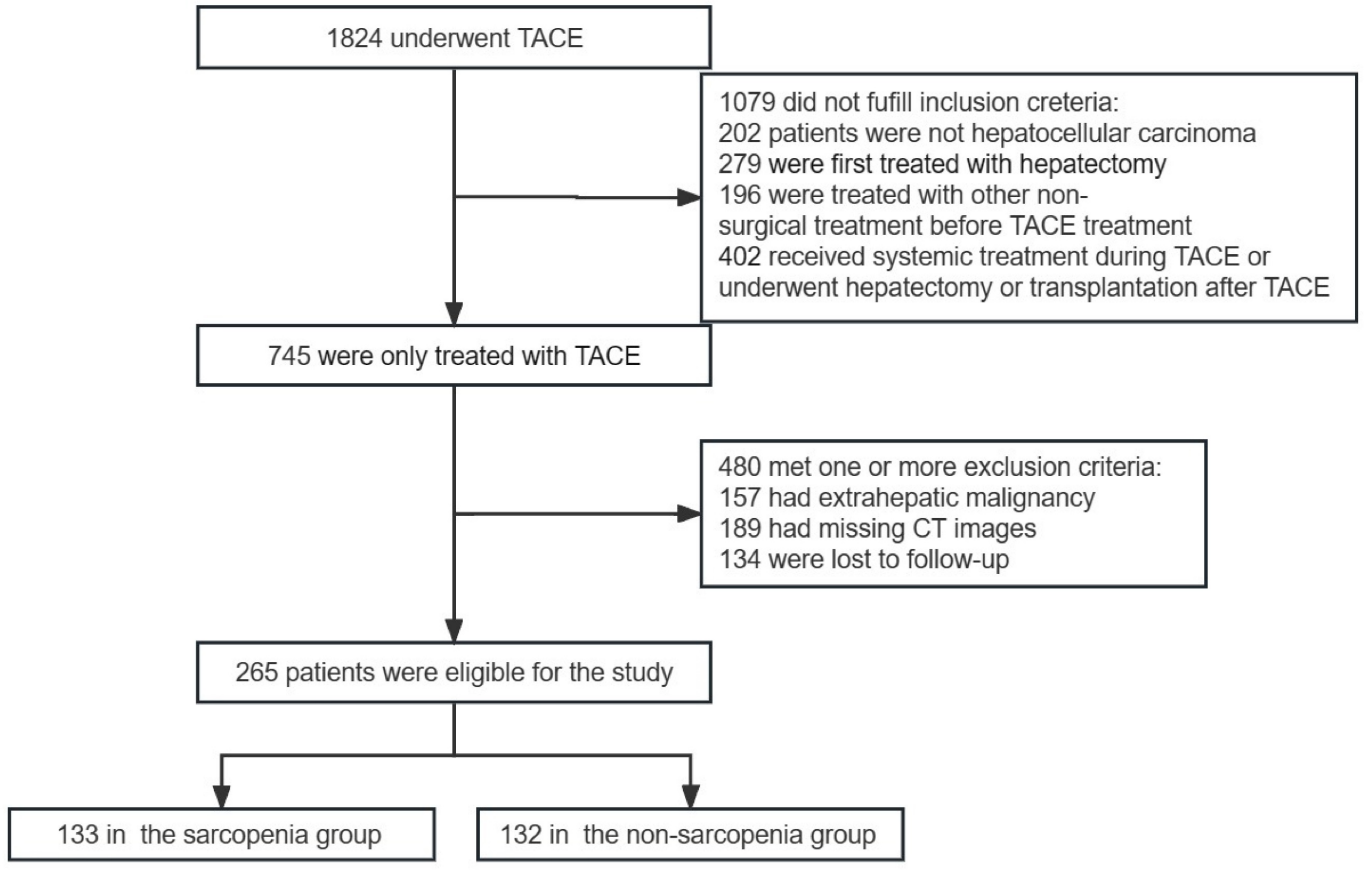
Criteria and flowchart for inclusion and exclusion of the study.

We conducted a retrospective analysis of clinical data from 1,824 HCC patients who were treated with TACE at Wuhan union hospital between April 2014 and February 2021. Based on our inclusion and exclusion criteria, 265 patients were enrolled in this study, including 133 in the sarcopenia group and 132 in the non-sarcopenia group (Figure 1). The study's inclusion criteria comprised a confirmed diagnosis of HCC in accordance with the CNLC (Chinese liver cancer) guidelines^19^, with the initial treatment for HCC being TACE due to the absence of indications for hepatectomy, liver transplantation, or ablation, or due to the patient's refusal of surgery. Additionally, patients included in the study had not undergone any other non-surgical treatments before TACE, had not received systemic treatments during the TACE period, and had not undergone subsequent surgical procedures such as hepatectomy or liver transplantation following TACE treatment. Exclusion criteria encompassed patients with malignant tumors or liver metastasis other than HCC, those with missing imaging data, such as incomplete computed tomography (CT) examination data, and individuals who were lost to follow-up after TACE treatment.

### Measurement of sarcopenia

We used L3 as the bony landmark and measured the total skeletal muscle area (SMA) of all skeletal muscles (including the psoas major, psoas minor, quadratus lumborum, erector spine, transverse abdominis, external and internal obliques) at the L3 level on pre-TACE CT plain scans of all eligible patients (Figure S1). During the measurement process, the average CT value (in Hounsfield units) of all muscles within the measurement range was calculated and recorded. Tissue with CT values ranging from 29 to 150 HU was considered as muscle. Subsequently, the L3-SMA was standardized to L3-SMI (SMA/height^2^) based on each patient's height. Using the sarcopenia diagnostic criteria recommended by the Japan Society of Hepatology (JSH) (L3-SMI < 42 cm^2^/m^2^ for males and L3-SMI < 38 cm^2^/m^2^ for females)^20^, patients were divided into the sarcopenia and non-sarcopenia groups. We defined low muscle attenuation (LMA) as a muscle CT value lower than 41 HU and a body mass index (BMI) < 25 kg/m^2^ or a muscle CT value lower than 33 HU and BMI ≥ 25 kg/m^2^, indicating skeletal muscle fat infiltration. All measurements were performed by two senior physicians, and the average value of the two measurements was selected. The operators were blinded to each other's measurements.

### TACE procedure

The femoral artery was punctured using the Seldinger technique, and angiography was performed to identify the tumor-related situation and portal vein blood flow. A microcatheter was then super-selected into the tumor-feeding artery branch for embolization treatment. The embolic agents used mainly included iodized oil (5-20 mg) and drug-loaded microspheres (100-500 µm), and the chemotherapy drugs mainly included pirarubicin (10-80 mg). Additional embolic agents were added as needed until satisfactory embolization was achieved (Figure S2). After TACE, we paid attention to whether there was bleeding or hematoma at the puncture site and monitored for the syndrome of post-embolization. Appropriate symptomatic treatment was given as needed.

### Post-TACE follow-up

The participants underwent TACE and were followed up at 4-6 weeks after the initial procedure, and subsequently every 3 months. The follow-up involved liver imaging examination, in addition to laboratory tests that included blood routine, tumor markers (AFP), liver and kidney function, and coagulation function. If there was evidence of tumor enhancement or new lesions during the follow-up, participants received additional rounds of TACE. The follow-up period continued until the end of the study or until September 2022, whichever came first.

### Endpoints of observation

The primary endpoint of this study was OS during the follow-up after TACE, and the secondary endpoints were PFS, objective response rate (ORR), and disease control rate (DCR) at 3 and 6 months after TACE. OS was defined as the time interval from the first TACE treatment to death, and for patients who were still alive as of September 2022, the end time was the last follow-up time. PFS was defined as the time interval from the first TACE treatment to disease progression. ORR was the proportion of patients who experienced either a partial response or a complete response to treatment. DCR was the proportion of patients who experienced either a partial response, stable disease, or complete response to treatment. Local tumor response was evaluated using the mRECIST criteria after TACE^21^ (Figure S3).

### Statistical analysis

All data analyses were performed using R 3.3.3 and SPSS 24.0 statistical software, and GraphPad Prism 8 was used to draw relevant statistical graphs. Continuous variables in the patients' clinical characteristics were expressed as the mean ± standard deviation and compared using t-tests, while categorical variables were expressed as proportions and compared using chi-squared tests or Fisher's exact tests. Kaplan-Meier curves were used to calculate OS and PFS for the two groups of patients, and differences were compared using the Log-rank test. Cox regression analysis was used to identify the risk factors affecting OS and PFS after TACE, and factors with P < 0.10 in the univariate analysis were included in the multivariate analysis. Receiver operating characteristic (ROC) curves were plotted, and the area under the curve (AUC) was calculated to determine the optimal cutoff value for maximal tumor diameter. Meaningful risk factors were then selected to construct a prognostic grading system and draw a nomogram to predict the probability of OS and PFS in different risk groups. A p-value less than 0.05 was considered statistically significant.

## Results

### Baseline Characteristics of Patients

A total of 265 HCC patients who underwent TACE from April 2014 to February 2021 were included in this study, with 133 in the sarcopenia group and 132 in the non-sarcopenia group. The sarcopenia group had a higher average age than the non-sarcopenia group (57.9 ± 13.2 vs 54.7 ± 10.4, *P*=0.026); a lower average weight (60.0 ± 8.4 vs 66.9 ± 8.9, *P*<0.001); a lower average BMI (21.0 ± 3.2 vs 23.6 ± 2.8, *P*<0.001); and a lower average L3-SMA (104.2 ± 17.1 vs 132.8 ± 17.8, *P*<0.001). Additionally, there were significant differences between the two groups in L3-SMI, muscle attenuation, maximal tumor diameter, albumin, creatinine, albumin to globulin ratio (AAR), platelet to lymphocyte ratio (PLR), and neutrophil to lymphocyte ratio (NLR) (Table 1).

### Efficacy of TACE at 3 and 6 months after TACE in sarcopenia and non-sarcopenia groups

The tumor response to local treatment after TACE was evaluated according to the mRECIST criteria at 3 and 6 months after TACE (Table 2). 3 months after TACE, there was a significant difference in the efficacy evaluation between the two groups (*P*<0.001). In the sarcopenia group, only 7 patients (5.3%) were evaluated as CR, while in the non-sarcopenia group, 20 patients (14.5%) were evaluated as CR. The ORRs were 59.8% and 35.3% (*P*<0.001) and the DCRs were 77.3% and 72.9% (*P*=0.414) in the non-sarcopenia and sarcopenia groups, respectively. The efficacy at 6 months after TACE also showed a statistically significant difference (*P*=0.001). The ORRs were 51.5% and 28.6% (*P*<0.001) and the DCRs were 64.4% and 46.6% (*P*=0.004) in the non-sarcopenia and sarcopenia groups, respectively.

### Comparison of OS after TACE in sarcopenia and non-sarcopenia groups

**Figure 2:**
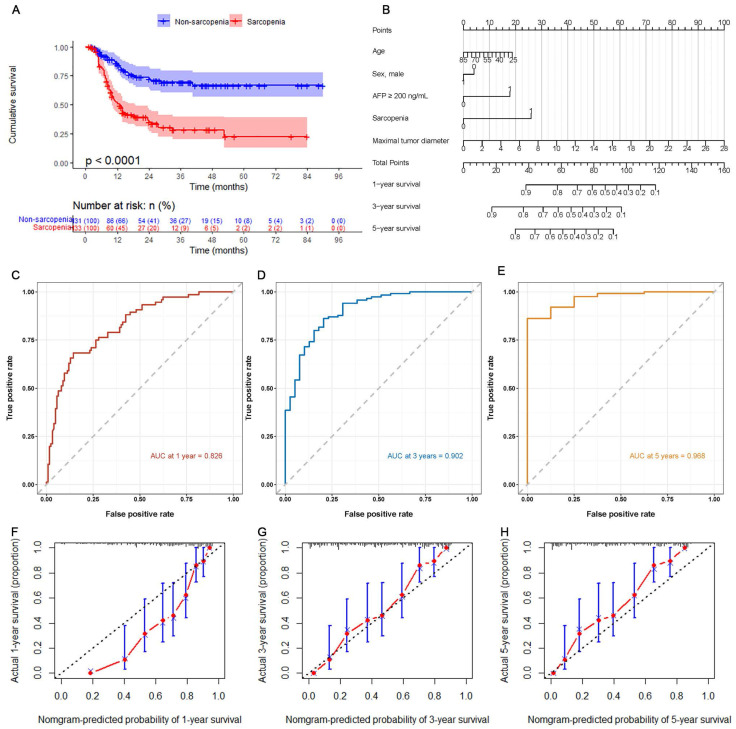
** Predictive value of sarcopenia for post-TACE patient OS.** (A) Kaplan-Meier curves for OS of the sarcopenia and non-sarcopenia groups. (B) Nomogram of the predictive model for post-TACE OS based on Cox multivariate analysis. (C-E) ROC curves for assessing the accuracy of the predictive model. (F-H) Calibration plots for evaluating the calibration of the predictive model.

The Kaplan-Meier curves showed that the cumulative survival rate in the non-sarcopenia group was significantly higher than that in the sarcopenia group (HR=3.319, 95%CI: 2.283-4.824, Log-rank *P*<0.001) (Figure 2). The median follow-up time was 13.0 months (7.0-28.9). The cumulative rate of free of death in the non-sarcopenia group at 12, 36, and 60 months after TACE were 83.4%, 68.8%, and 66.2%, respectively, while those in the sarcopenia group were 52.2%, 28.3%, and 22.6%, respectively. Cox univariate regression analysis was performed on the patients' clinical data, and variables with P<0.10 in the univariate analysis were included in the Cox multivariate regression analysis after excluding confounding factors. The results showed that sarcopenia (HR=2.379, 95%CI: 1.552-3.647, *P*<0.001), AFP ≥ 200 ng/mL (HR=2.083, 95%CI: 1.372-3.161, *P*=0.001), and maximal tumor diameter (HR=1.153, 95%CI: 1.108-1.199, *P*<0.001) were independent risk factors for OS (Table 3). A nomogram was constructed based on the results of the multivariate analysis, and the predictive performance of the nomogram was evaluated using ROC curves and calibration plots. The ROC analysis demonstrated that the predictive accuracies of the model were high for all three-time points (1, 3, and 5 years), with AUC values of 0.826, 0.902, and 0.968, respectively. The calibration analysis indicated that the model had a good predictive performance.

### Comparison of PFS after TACE in sarcopenia and non-sarcopenia groups

**Figure 3:**
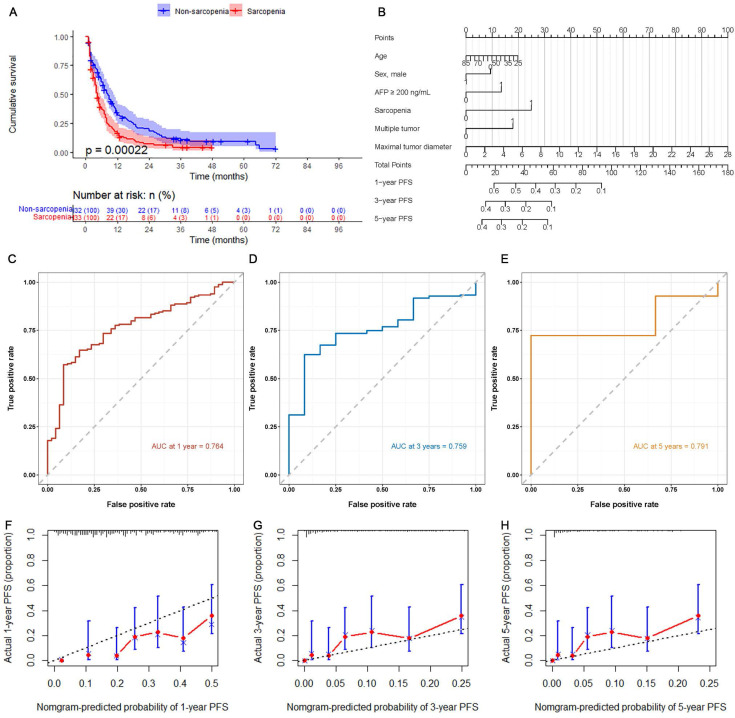
** Predictive value of sarcopenia for post-TACE patient PFS.** (A) Kaplan-Meier curves for PFS of the sarcopenia and non-sarcopenia groups. (B) Nomogram of the predictive model for post-TACE PFS based on Cox multivariate analysis. (C-E) ROC curves for assessing the accuracy of the predictive model. (F-H) Calibration plots for evaluating the calibration of the predictive model.

The Kaplan-Meier curves showed that the cumulative PFS in the non-sarcopenia group was significantly higher than that in the sarcopenia group (HR=0.631, 95%CI: 0.486-0.820, Log-rank *P*<0.001) (Figure 3). The median follow-up time was 5.5 months (IQR: 2.0-11.3). The cumulative rate of free of progression in the non-sarcopenia group at 12, 36, and 60 months after TACE were 34.6%, 20.1%, and 9.4%, respectively, while those in the sarcopenia group were 16.9%, 6.3%, and 4.2%, respectively. Cox univariate regression analysis was performed on the patients' clinical data, and variables with *P*<0.10 in the univariate analysis were included in the Cox multivariate regression analysis after excluding confounding factors. The results showed that sarcopenia (HR=1.577, 95%CI: 1.181-2.106, *P*=0.002), AFP ≥ 200 ng/mL (HR=1.361, 95%CI: 1.024-1.809, P=0.033), multifocal tumor (HR=1.423, 95%CI: 1.066-1.899, *P*=0.017), and maximal tumor diameter (HR=1.075, 95%CI: 1.041-1.110, *P*<0.001) were independent risk factors for PFS (Table 4). A nomogram was constructed based on the results of the multivariate analysis, and the predictive performance of the nomogram was evaluated using ROC curves and calibration plots. The ROC analysis demonstrated that the predictive accuracies of the model were high for all three time points (1, 3, and 5 years), with AUC values of 0.764, 0.759, and 0.791, respectively. The calibration plots also showed good predictive performance of the nomogram.

### Construction of a prognostic risk grading system

**Figure 4:**
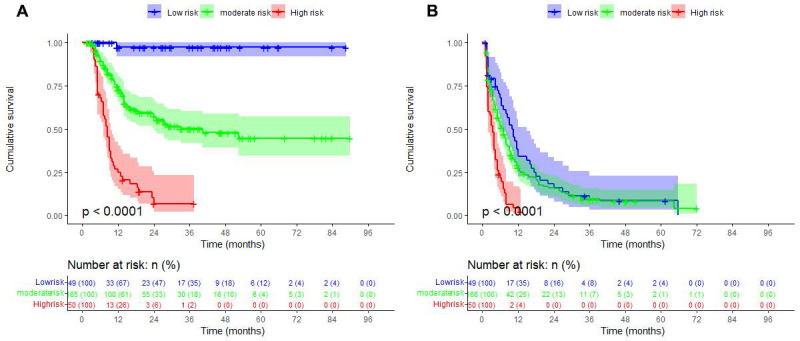
** Evaluation of the prognostic risk grading system using Kaplan-Meier curves.** (A) Significant differences in OS among patients with different risk levels. (B) Significant differences in PFS among patients with different risk levels.

ROC analysis was performed on the maximal tumor diameter to determine the optimal cutoff value for survival, which was 8.9 cm (Figure S4). A TACE prognostic grading system was constructed based on whether the patient had AFP ≥ 200 ng/mL, maximal tumor diameter ≥ 8.9 cm, and sarcopenia (Figure 4). The low-risk group was defined as having no risk factors, the medium-risk group was defined as having 1 or 2 risk factors, and the high-risk group was defined as having 3 risk factors. The Kaplan-Meier curves showed that the cumulative rate of free of death in the low-risk group alive at 12, 36, and 60 months after TACE were 97.2%, 85.3%, and 76.1%, respectively, while those in the medium-risk group were 72.1%, 49.8%, and 44.2%, respectively, and those in the high-risk group were 24.7%, 10.3%, and 6.9%, respectively. There were significant differences in OS among the different risk groups (*P*<0.001). The Kaplan-Meier curves showed that the cumulative rate of free of progression in the low-risk group at 12, 36, and 60 months after TACE were 38.5%, 11.3%, and 8.5%, respectively, while those in the medium-risk group were 27.8%, 9.4%, and 8.1%, respectively, and those in the high-risk group were 4.4%, 2.2%, and 0%, respectively. There were significant differences in PFS among the different risk groups (*P*<0.001).

## Discussion

Previous studies have shown that sarcopenia is associated with poor clinical outcomes in HCC patients receiving various treatments^22^, ^23^. In this study, Cox regression analysis showed that the cumulative survival rates (OS and PFS) of the non-sarcopenia group were significantly higher than those of the sarcopenia group, and sarcopenia was an independent risk factor for poor prognosis in patients. Currently, there was a lack of standardized diagnostic criteria for sarcopenia^24^. In this study, we utilized CT to quantify skeletal muscle mass according to the diagnostic criteria recommended by the Japan Society of Hepatology (JSH)^20^. CT served as a standard procedure for precisely measuring skeletal muscle mass, providing an objective and comprehensive assessment of the patient's nutritional and metabolic status^25^. Furthermore, as CT was routinely conducted prior to TACE, it enabled an accurate evaluation of the patient's baseline sarcopenia^26^.

Patients with hepatocellular carcinoma often experience additional complications such as ascites and edema, which can result in abnormalities in renal function, immune function, and protein metabolism^27^. This can lead to a decline in nutritional status and changes in body composition, particularly in those with advanced liver cancer. Furthermore, the combination of hepatocellular carcinoma with cirrhosis of the liver can lead to increased protein catabolism, while the presence of portal hypertensive gastrointestinal disease can result in reduced nutrient intake^26^. In addition, due to the huge consumption of the body caused by tumor burden, many liver cancer patients suffered from sarcopenia^28^. And with the progression of the tumor and the treatments such as hepatectomy and chemotherapy, sarcopenia often worsens^22^. In turn, worsening sarcopenia made patients more susceptible to chemotherapy toxicity, thereby weakening treatment effectiveness^29^. Currently, there have been multiple studies indicating that sarcopenia is an independent risk factor for HCC surgery, liver transplantation, radiofrequency ablation, and systemic treatment, and may be related to an increased risk of hepatic encephalopathy, post-transplant mortality, infection, treatment response, and reduced quality of life, or even decreased patient survival and increased treatment-related mortality^30^-^33^.

However, the impact of sarcopenia on the prognosis of HCC patients receiving TACE treatment has not been widely evaluated. There was a study that investigated the prognosis of HCC patients who underwent TACE treatment. The monthly change in the surrogate of sarcopenia, represented by the psoas muscle index (PMI), was found to be significantly associated with a decrease in OS^14^; Kobayashi et al. reported that rapid decline in skeletal muscle mass predicted poor prognosis in patients undergoing transcatheter arterial therapy for HCC^15^. However, the impact of whether patients have sarcopenia before TACE on long-term poor outcomes was still controversial. Kobayashi et al. and Fujita et al. reported that there was no significant association between baseline muscle mass and clinical outcomes^15^, ^30^, which contradicted our findings. This might be due to different definitions of sarcopenia in different studies, and sarcopenia was also related to different races. On the other hand, differences in the severity of disease in the included patients may also lead to differences in the results. In addition, some studies were consistent with our results. Loosen et al. and Dodson et al. showed that pre-TACE sarcopenia was an independent risk factor for poor outcomes^14^, ^34^. Previous research has shown that loss of muscle mass is a significant prognostic factor in patients with hepatocellular carcinoma undergoing transarterial embolization therapy. It was associated with increased mortality, without impairing the safety of the locoregional treatment^35^. Given the severity and complexity of liver cancer, patients with sarcopenia are difficult to reverse and will only worsen, and even some patients without sarcopenia before TACE may develop sarcopenia as the disease progresses. Therefore, we suspect that pre-TACE sarcopenia will have a significant impact on post-TACE clinical outcomes, which is consistent with our research results. In addition, some studies have reported that pre-TACE sarcopenia was not related to treatment response and the risk of perioperative complications^35^, which was not inconsistent with our research results. This suggested that the impact of sarcopenia on patient clinical outcomes was not achieved by affecting the efficacy of local treatment, but by affecting the general physical status of patients.

We also found that baseline AFP ≥ 200ng/mL was an independent risk factor for poor prognosis. AFP has been proven to be a key tumor marker for HCC and was significantly correlated with patient prognosis^36^, ^37^. In addition, after TACE, the risk of death in patients with continuous AFP increase was about 10 times that of patients with rapid AFP decrease, and there was also a significant correlation between baseline AFP level and post-TACE AFP increase^38^. In our study, maximal tumor diameter was also an independent risk factor affecting patient prognosis. It has been reported that TACE was not effective for patients with huge tumors^39^. In a study of TACE combined with ablation, maximal tumor diameter was an important prognostic factor for OS and PFS^40^. Severe tumor burden significantly reduced the long-term survival of patients, and in this study, the multifocal tumor was confirmed to be an independent risk factor for PFS. Consistent with our study, Seung Baek Hong et al. believed that multifocal tumor was an important predictor of HCC microvascular invasion^41^, which is often related to tumor metastasis and invasion.

In addition to TACE, sarcopenia significantly influenced the prognosis of other hepatocellular carcinoma treatments, including hepatectomy, systemic therapy, and liver transplantation. However, prevalent prognostic scoring systems such as ALBI, MELD, and Child-Pugh did not consider patients' nutritional status. As a result, a prognostic assessment system that incorporated sarcopenia would serve as a valuable tool for predicting outcomes. Based on the results of multivariate analysis of OS and PFS, we constructed a TACE postoperative prognosis risk assessment system based on baseline AFP ≥ 200ng/mL, maximal tumor diameter ≥ 8.9 cm, and sarcopenia. We found that the prognosis of the high-risk group was significantly worse than other patients. Therefore, patients with multiple risk factors for poor outcomes should be closely monitored during treatment. Among these three risk factors, improving the patient's sarcopenia is crucial, and improving or even reversing the patients' sarcopenia can improve their short-term and long-term prognosis. International guidelines on sarcopenia released in 2018 strongly recommend resistance-based training and conditionally recommend increasing protein and calorie intake and supplementing protein if necessary^42^. Hurst et al. recommended a twice-weekly exercise regimen for treating sarcopenia, involving combined upper and lower body exercises, performing 1-3 sets at high intensity, and repeating each set 6-12 times^43^, and this exercise regimen has been shown to be effective in treating sarcopenia. The importance of sarcopenia as a prognostic factor in various diseases is well acknowledged, but further in-depth studies are needed to improve sarcopenia. In addition to promoting increased physical activity and lifestyle modifications, there should be a focus on the development of pharmaceutical interventions. The creation of targeted medications has the potential to greatly enhance patient outcomes.

Our study also has some limitations. First, this study is retrospective, and there is therefore selection bias due to the nature of the study design. Second, in the diagnosis of sarcopenia, muscle quality is crucial^44^. We only used CT images to diagnose sarcopenia and did not evaluate muscle quality, which may lead to errors in the diagnosis of sarcopenia. The diagnostic criteria we used are based on Asian populations, and different diagnostic criteria may yield different results. In addition, we only evaluated the sarcopenia of pre-TACE patients and did not study the progression of sarcopenia in post-TACE patients. Some studies have reported that the progression of post-TACE sarcopenia was more predictive of patient prognosis^16^, so exploring the changes or progression of sarcopenia in post-TACE patients would have more clinical value.

## Conclusion

In conclusion, sarcopenia had excellent predictive value for the OS and PFS of post-TACE patients, and AFP ≥ 200ng/mL and maximal tumor diameter were also independent risk factors for poor prognosis. The prognosis risk grading system based on sarcopenia, AFP, and maximal tumor diameter had good guiding value for the prognosis of patients.

## Supplementary Material

Supplementary figures.

## Figures and Tables

**Table 1:** Baseline characteristics of the patients with and without sarcopenia.

Variables	All patients	Sarcopenia	Non-sarcopenia	P values
	(n = 265)	(n = 133)	(n = 132)	
**Demographic Characteristics**				
Age, years	56.3 ± 12.0	57.9 ± 13.2	54.7 ± 10.4	0.026
Gender, male	229 (86.4)	110 (82.7)	119 (90.2)	0.077
Gender, female	36 (13.6)	23 (17.3)	13 (9.8)	
Body weight, kg	63.5 ± 9.3	60.0 ± 8.4	66.9 ± 8.9	< 0.001
Height	1.68 ± 0.06	1.68 ± 0.06	1.68 ± 0.06	0.623
BMI, kg/m^2^	22.4 ± 3.3	21.0 ± 3.2	23.6 ± 2.8	< 0.001
**Etiology**				0.447
HBV	190 (71.7)	102 (76.7)	90 (68.2)	
HCV	18 (6.8)	7 (5.3)	11 (8.3)	
Alcohol	3 (1.1)	1 (0.8)	2 (1.5)	
Unknown	54 (20.4)	23 (17.3)	29 (22.0)	
**Comorbidity**				
Hypertension	24 (9.1)	11 (8.3)	13 (9.8)	0.655
Diabetes	10 (3.8)	5 (3.8)	5 (3.8)	0.99
Cirrhosis	189 (71.3)	96 (72.2)	97 (73.5)	0.811
**Laboratory Parameters**				
TBIL, μmol/L	19.7 ± 12.0	19.4 ± 11.5	20.1 ± 12.4	0.639
ALB, g/L	36.5 ± 5.1	35.9 ± 5.2	37.2 ± 4.8	0.037
ALT, U/L	57.6 ± 66.2	55.6 ± 41.4	59.8 ± 85.3	0.619
AST, U/L	75.5 ± 78.6	80.0 ± 73.8	70.6 ± 83.5	0.352
Creatinine, μmol/L	69.8 ± 23.9	65.3 ± 14.3	74.7 ± 30.5	0.002
PT, seconds	14.0 ± 1.3	14.0 ± 1.5	14.1 ± 1.5	0.195
INR	1.10 ± 0.13	1.09 ± 0.11	1.11 ± 0.15	0.181
Platelet count, 10^9^/L	166.4 ± 89.1	171.4 ± 90.5	161.2 ± 87.6	0.36
Neutrophil count, 10^9^/L	3.7 ± 2.1	3.9 ± 2.3	3.6 ± 1.7	0.182
Lymphocyte count, 10^9^/L	1.3 ± 1.3	1.2 ± 0.6	1.5 ± 1.7	0.083
AFP ≥ 200 ng/mL	126 (47.5)	69 (51.9)	57 (43.2)	0.156
**Radiographic Analysis**				
Ascites	68 (26.7)	24 (18.0)	37 (28.0)	0.054
L3 SMA, cm^2^	118.5 ± 22.6	104.2 ± 17.1	132.8 ± 17.8	< 0.001
L3 SMI, cm^2^/m^2^	41.7 ± 6.7	36.7 ± 4.3	46.7 ± 4.7	< 0.001
Muscle attenuation	133 (50.2)	82 (61.7)	51 (38.6)	< 0.001
Multiple tumor	154 (58.1)	75 (56.4)	79 (59.8)	0.568
Maximal tumor diameter, cm	9.5 ± 4.5	10.5 ± 4.6	8.6 ± 4.4	< 0.001
**Scoring and staging systems**				
** BCLC stage**				0.276
A	56 (21.1)	23 (17.3)	33 (25.0)	
B	96 (36.2)	46 (34.6)	50 (37.9)	
C	113 (42.6)	61 (45.9)	52 (39.4)	
**ECOG-PS**				0.189
0	178 (67.2)	95 (71.4)	83 (62.9)	
1	81 (30.6)	38 (28.6)	43 (32.6)	
2	6 (2.3)	5 (3.8)	1 (0.8)	
** Child-Pugh Class**				0.976
A	176 (66.4)	90 (67.7)	86 (65.2)	
B	78 (29.4)	40 (30.1)	38 (28.8)	
C	11 (4.2)	6 (4.5)	5 (3.8)	
** HAP classification**				0.457
A	14 (5.3)	5 (3.8)	9 (6.8)	
B	65 (24.5)	31 (23.3)	34 (25.8)	
C	87 (33.8)	42 (31.6)	45 (34.1)	
D	99 (37.4)	55 (41.3)	44 (33.3)	
AAR	1.18 ± 2.34	0.9 ± 0.8	1.5 ± 3.3	0.032
NLR	3.48 ± 2.64	3.9 ± 3.0	3.1 ± 3.0	0.011
PLR	151.3 ± 105.2	168.6 ± 122.2	133.3 ± 80.5	0.007
ALBI	-2.29 ± 0.49	-2.23 ± 0.50	-2.35 ± 0.48	0.076

Data presented as mean ± SD or number of patients (%) where appropriate. Abbreviation: BMI, body mass index; HBV, hepatitis B virus; HCV, hepatitis C virus; TBIL, total bilirubin; ALB, albumin; AST, aspartate aminotransferase; ALT, alanine aminotransferase; AFP, alpha-fetoprotein; SMA, skeletal muscle area; SMI, skeletal muscle Index; AAR, albumin to globulin ratio; NLR, neutrophil to lymphocyte ratio; PLR, platelet to lymphocyte ratio; HAP, hepatoma arterial-embolisation prognostic; ALBI, albumin bilirubin.

**Table 2:** Treatment response at 3 and 6 months after TACE in patients with and without sarcopenia.

	All patients (n = 265)	Sarcopenia (n = 133)	Non-sarcopenia (n = 132)	P values
**3-month**				< 0.001
CR	27 (10.1)	7 (5.3)	20 (15.2)	
PR	99 (37.4)	40 (30.1)	59 (44.7)	
SD	73 (27.5)	50 (37.6)	23 (17.4)	
PD	66 (24.9)	36 (27.1)	30 (22.7)	
**6-momth**				0.001
CR	25 (9.4)	6 (4.5)	19 (14.4)	
PR	81 (30.6)	32 (24.1)	49 (37.1)	
SD	41 (15.5)	24 (18.0)	17 (12.9)	
PD	118 (44.5)	71 (53.4)	47 (35.6)	

Data presented number of patients (%). Abbreviation: CR, complete response; PR, partial response; SD, stable disease; PD, progressive disease.

**Table 3:** Univariable and multivariable analysis of factors associated with OS

Variables	Univariable analysis			Multivariable analysis		
	HR	95% CI	P value	HR	95% CI	P value
Age, years	1.011	1.000-1.030	0.137	-	-	-
Gender, male	1.192	0.668-2.128	0.553	-	-	-
BMI, kg/m^2^	0.902	0.844-0.965	0.003	-	-	-
Cirrhosis, yes	0.836	0.558-1.252	0.385	-	-	-
TBIL, μmol/L	1.007	0.993-1.022	0.299	-	-	-
ALB, g/L	0.963	0.927-1.000	0.049	-	-	-
ALT, U/L	1.002	0.998-1.005	0.323	-	-	-
AST, U/L	1.003	1.002-1.005	< 0.001	-	-	-
Creatinine, μmol/L	0.986	0.974-0.998	0.022	-	-	-
INR	0.392	0.095-1.617	0.195	-	-	-
Platelet count, 10^9^/L	1.003	1.001-1.005	0.002	-	-	-
AFP ≥ 200 ng/mL, yes	2.472	1.631-3.745	< 0.001	2.083	1.372-3.161	0.001
Ascites, yes	1.370	0.892-2.103	0.150	-	-	-
Sarcopenia, yes	3.437	2.264-5.217	< 0.001	2.379	1.552-3.647	< 0.001
Muscle attenuation, yes	1.555	1.066-2.267	0.022	-	-	-
Multifocal tumor, yes	0.704	0.485-1.021	0.064	-	-	-
Maximal tumor diameter, cm	1.185	1.142-1.230	< 0.001	1.153	1.108-1.199	< 0.001
BCLC C, yes	4.920	3.302-7.331	< 0.001	-	-	-
Child-Pugh A, yes	0.679	0.458-1.007	0.054	-	-	-
HAP D, yes	3.245	2.206-4.786	< 0.001	-	-	-
AAR	0.543	0.356-0.828	0.005	-	-	-
NLR	0.918	1.032-1.149	0.002	-	-	-
PLR	1.004	1.002-1.005	< 0.001	-	-	-
ALBI	1.505	1.039-2.179	0.031	-	-	-

Abbreviation: HR, hazard ratio; CI, confidence interval; BMI, body mass index; TBIL, total bilirubin; ALB, albumin; AST, aspartate aminotransferase; ALT, alanine aminotransferase; AFP, alpha-fetoprotein; HAP, hepatoma arterial-embolisation prognostic; AAR, albumin to globulin ratio; NLR, neutrophil to lymphocyte ratio; PLR, platelet to lymphocyte ratio; ALBI, albumin bilirubin.

**Table 4:** Univariable and multivariable analysis of factors associated with PFS

Variables	Univariable analysis			Multivariable analysis		
	HR	95% CI	P value	HR	95% CI	P value
Age, years	0.990	0.979-1.001	0.084	-	-	-
Gender, male	1.008	0.693-1.466	0.967	-	-	-
BMI, kg/m^2^	0.978	0.938-1.020	0.305	-	-	-
Cirrhosis, yes	1.089	0.817-1.452	0.560	-	-	-
TBIL, μmol/L	0.993	0.982-1.005	0.250	-	-	-
ALB, g/L	0.987	0.962-1.012	0.296	-	-	-
ALT, U/L	1.000	0.998-1.003	0.814	-	-	-
AST, U/L	1.001	0.999-1.002	0.324	-	-	-
Creatinine, μmol/L	0.998	0.992-1.004	0.443	-	-	-
INR	0.368	0.141-0.963	0.041	-	-	-
Platelet count, 10^9^/L	1.002	1.000-1.003	0.008	-	-	-
AFP ≥ 200 ng/mL, yes	1.432	1.087-1.887	0.011	1.361	1.024-1.809	0.033
Ascites, yes	1.089	0.817-1.452	0.560	-	-	-
Sarcopenia, yes	1.635	1.258-2.125	< 0.001	1.577	1.181-2.106	0.002
Muscle attenuation, yes	1.469	1.123-1.896	0.005	-	-	-
Multifocal tumor, yes	1.250	0.961-1.625	0.096	1.423	1.066-1.899	0.017
Maximal tumor diameter, cm	1.061	1.032-1.091	< 0.001	1.075	1.041-1.110	< 0.001
BCLC C, yes	2.127	1.620-2.792	< 0.001	-	-	-
Child-Pugh A, yes	0.912	0.680-1.222	0.536	-	-	-
HAP D, yes	1.538	1.156-2.046	0.003	-	-	-
AAR	0.901	0.791-1.027	0.118	-	-	-
NLR	1.016	0.973-1.062	0.464	-	-	-
PLR	1.001	1.000-1.003	0.047	-	-	-
ALBI	1.100	0.856-1.413	0.459	-	-	-

Abbreviation: HR, hazard ratio; CI, confidence interval; BMI, body mass index; TBIL, total bilirubin; ALB, albumin; AST, aspartate aminotransferase; ALT, alanine aminotransferase; AFP, alpha-fetoprotein; HAP, hepatoma arterial-embolisation prognostic; AAR, albumin to globulin ratio; NLR, neutrophil to lymphocyte ratio; PLR, platelet to lymphocyte ratio; ALBI, albumin bilirubin.
