# Supplementary material for: Impact of Sarcopenia on Prognosis in Primary Hepatocellular Carcinoma Patients Treated with Transcatheter Arterial Chemoembolization: A Single Center Retrospective Study
Source: J Cancer. 2024 Feb 4;15(7):1837–47. doi: 10.7150/jca.92976 (PMC10905400; doi:10.7150/jca.92976)
Supplement: Supplementary file 1 — Supplementary figures. [file jcav15p1837s1.pdf]

Impact of Sarcopenia on Prognosis in Primary Hepatocellular Carcinoma Patients Treated with Transcatheter Arterial Chemoembolization: A Single Center Retrospective Study

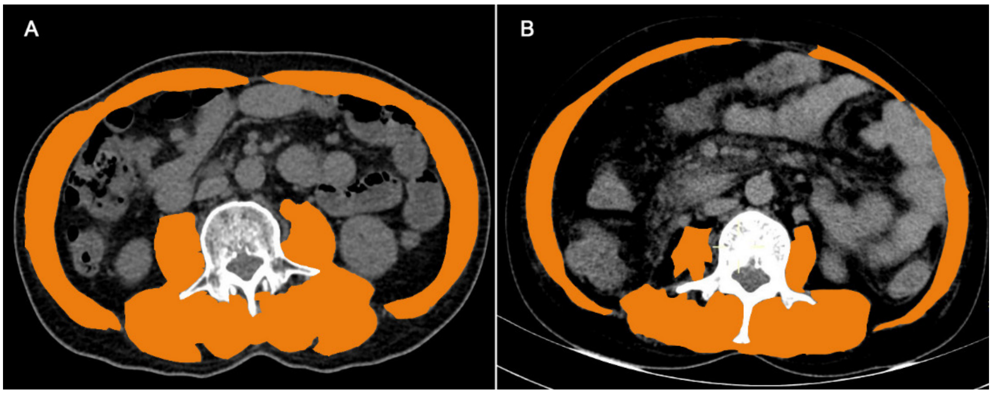

Figure S1. Representative images of measuring the skeletal muscle area at the L3 level on preoperative CT scan for patients. (A) A patient without sarcopenia. (B) A patient with sarcopenia.

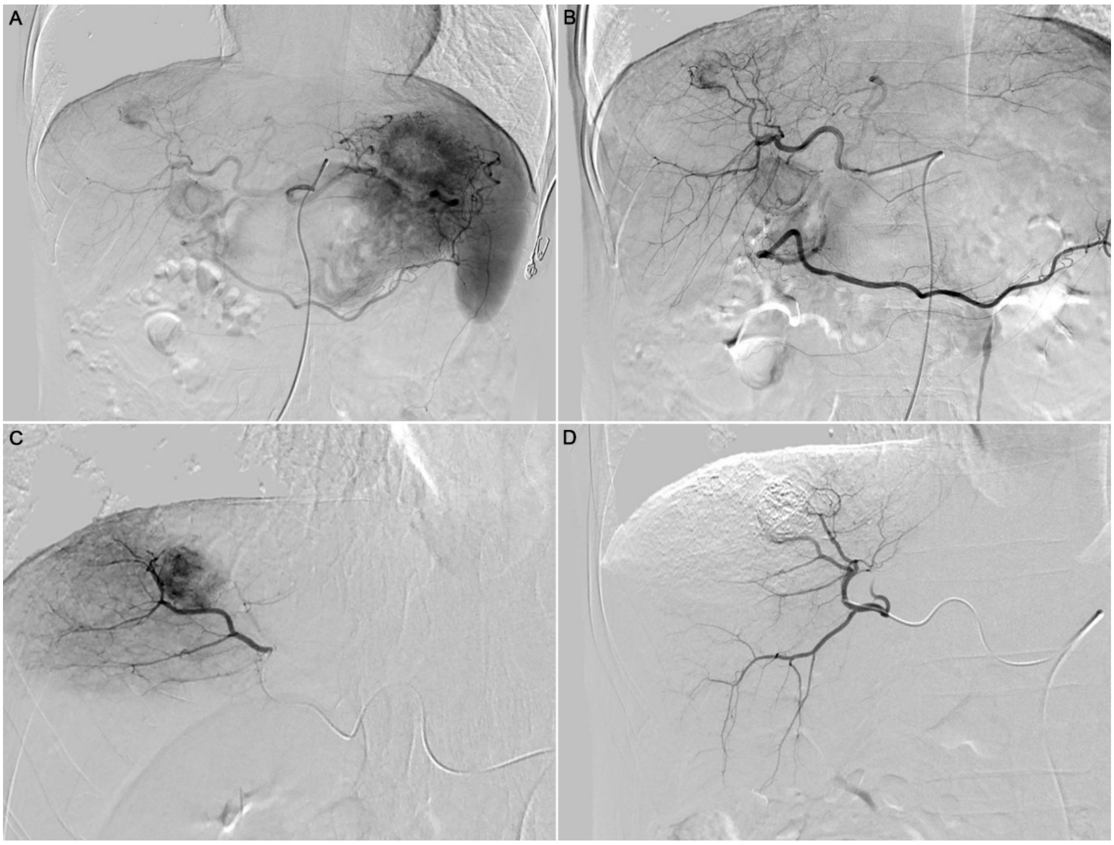

Figure S2. Representative images of TACE procedure. (A) Hepatic angiography showing abnormal staining mass in the right lobe of the liver. (B-C) The microcatheter is advanced to the tumor-feeding artery. (D) Repeat angiography after embolization shows no staining in the mass.

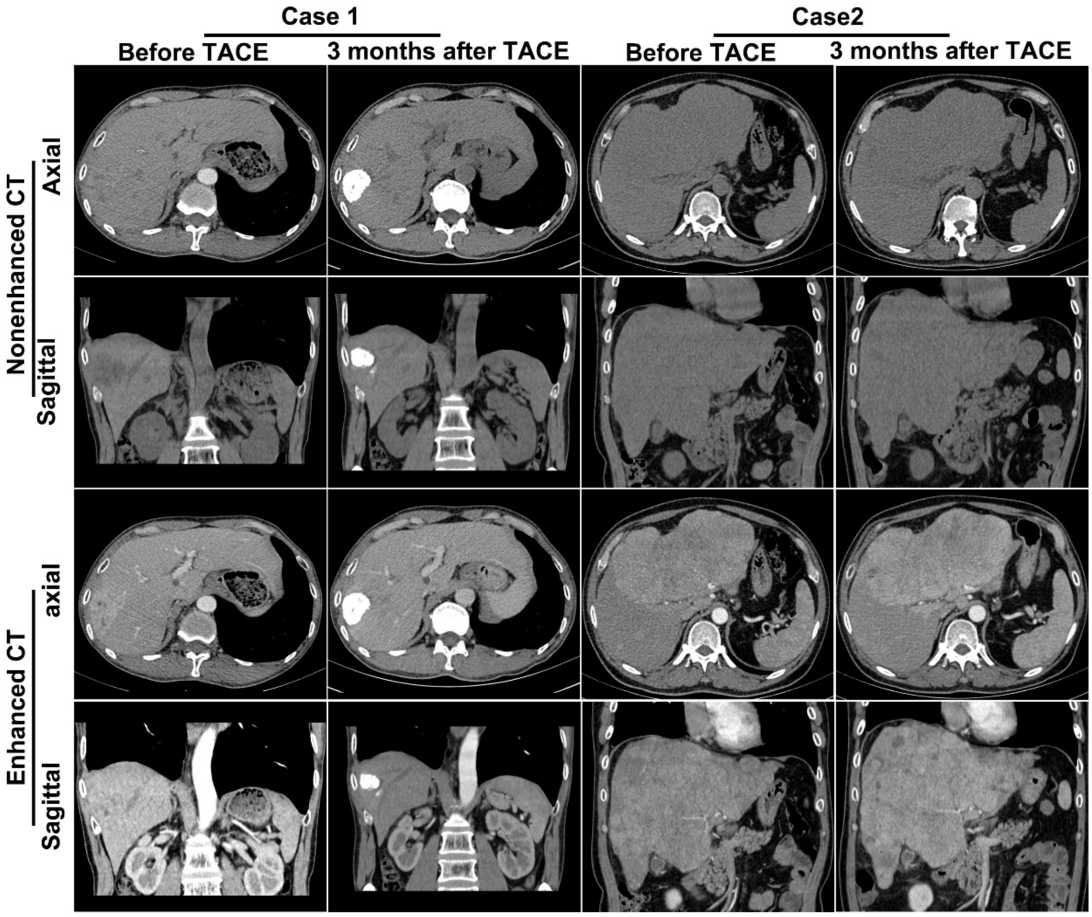

Figure S3. Representative images of mRECIST evaluation for patients.

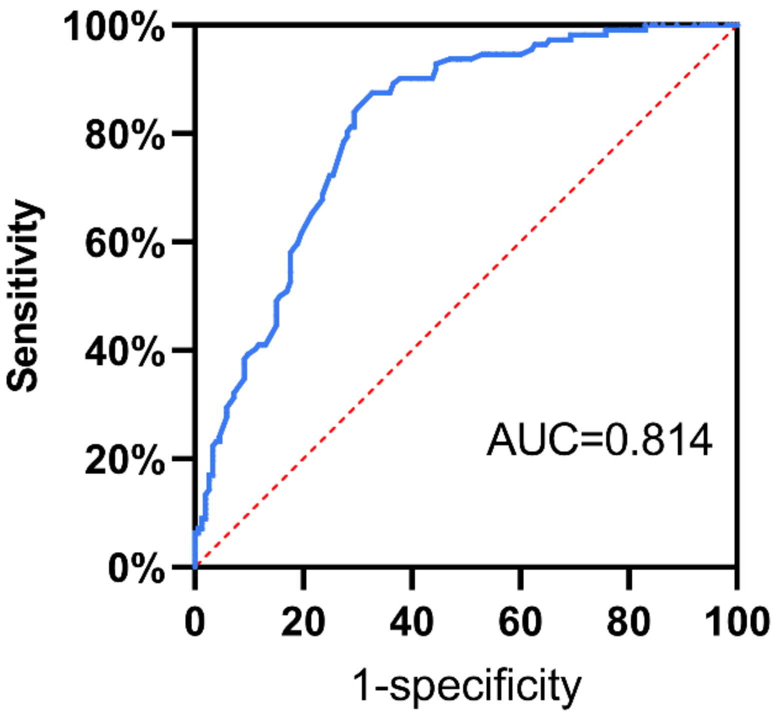

Figure S4. Performing ROC analysis on the maximal tumor diameter with respect to survival.
